# Andrographolide induces Nrf2 and heme oxygenase 1 in astrocytes by activating p38 MAPK and ERK

**DOI:** 10.1186/s12974-016-0723-3

**Published:** 2016-09-23

**Authors:** Siew Ying Wong, Michelle G. K. Tan, Peter T. H. Wong, Deron R. Herr, Mitchell K. P. Lai

**Affiliations:** 1 Department of Pharmacology, Yong Loo Lin School of Medicine, National University of Singapore, Unit 09-01, Centre for Translational Medicine (MD6), 14 Medical Drive, Kent Ridge, 117599 Singapore; 2 Department of Clinical Research, Singapore General Hospital, Outram, Singapore

**Keywords:** Andrographolide, Nrf2, Heme oxygenase 1, Astrocyte, Antioxidant response, Mitogen-activated protein kinases

## Abstract

**Background:**

Andrographolide is the major labdane diterpenoid originally isolated from *Andrographis paniculata* and has been shown to have anti-inflammatory and antioxidative effects. However, there is a dearth of studies on the potential therapeutic utility of andrographolide in neuroinflammatory conditions. Here, we aimed to investigate the mechanisms underlying andrographolide’s effect on the expression of anti-inflammatory and antioxidant heme oxygenase-1 (HO-1) in primary astrocytes.

**Methods:**

Measurements of the effects of andrograholide on antioxidant HO-1 and its transcription factor, Nrf2, include gene expression, protein turnover, and activation of putative signaling regulators.

**Results:**

Andrographolide potently activated Nrf2 and also upregulated HO-1 expression in primary astrocytes. Andrographolide’s effects on Nrf2 seemed to be biphasic, with acute (within 1 h) reductions in Nrf2 ubiquitination efficiency and turnover rate, followed by upregulation of Nrf2 mRNA between 8 and 24 h. The acute regulation of Nrf2 by andrographolide seemed to be independent of Keap1 and partly mediated by p38 MAPK and ERK signaling.

**Conclusions:**

These data provide further insights into the mechanisms underlying andrographolide’s effects on astrocyte-mediated antioxidant, and anti-inflammatory responses and support the further assessment of andrographolide as a potential therapeutic for neurological conditions in which oxidative stress and neuroinflammation are implicated.

**Electronic supplementary material:**

The online version of this article (doi:10.1186/s12974-016-0723-3) contains supplementary material, which is available to authorized users.

## Background

Oxidative stress goes hand in hand with inflammation and their underlying mechanisms are inextricably interconnected [1]. Growing evidence suggest that oxidative stress and neuroinflammation underpin a diverse range of CNS diseases, including stroke, traumatic brain injury, multiple sclerosis, Alzheimer’s, Parkinson’s, and other neurodegenerative diseases [2–5]. The brain is particularly vulnerable to oxidative stress due to an abundance of iron and polyunsaturated fatty acids which are susceptible to lipid peroxidation [6, 7], thus underscoring the importance of maintaining redox balance for normal brain functioning. One mechanism by which the brain defends itself against oxidative insults is via the upregulation of antioxidant molecules. Interestingly, oxidative stress strongly induces expression of heme oxygenase-1 (HO-1) in the CNS, a system not actively involved in red blood cell metabolism [8]. Indeed, while HO-1’s primary function is the catalysis of heme, leading to the eventual formation of antioxidant bilirubin, carbon monoxide, and ferrous iron (Fe^2+^), and in the process, prevents heme-mediated free radical production [9], HO-1 is also found to have anti-neuroinflammatory and neuroprotective properties in the CNS [8, 10]. Furthermore, HO-1 upregulation has been reported to be a mechanism by which certain isoflavone metabolites protect astrocytes from hydrogen peroxide-induced reactive oxygen species [11]. Together with their well-recognized roles in facilitating neuronal trophic support, biochemical homeostasis, blood brain barrier integrity, response to neuroinflammatory signals and scar formation, astrocytes play a critical role in redox homeostasis and are the major source of antioxidant molecules and enzymes which protect them and the neurons they support from oxidative stresses [12, 13]. The critical involvement of astrocytes in neuroinflammation and oxidative stress protection as well as the pathogenicity of astrocyte dysregulation in various CNS diseases [14–16] gave research impetus to discover and characterize novel anti-neuroinflammatory/antioxidant therapeutics with efficacy on astrocytes. Besides the aforementioned work on isoflavone metabolites [11], a wide range of other bioactive molecules have been studied. Of these, andrographolide is a labdane diterpenoid derived from the herbaceous *Andrographis paniculata*, which has been traditionally used in Asia to treat a variety of ailments, including fever, cough, tuberculosis, snake bites, respiratory tract, and urinary tract infections [17, 18]. Andrographolide has been reported to exhibit anti-inflammatory and antioxidant activities in peripheral tissues [19]. Furthermore, we have previously shown that the brain-penetrant andrographolide attenuates IL-1β or lipopolysaccharide-stimulated upregulation of the C–C and C–X–C chemokines in the brain cortex as well as in cultured astrocytes [20, 21]. However, while the efficacy of andrographolide in reducing oxidative stress in the CNS has been demonstrated in several studies [22–24], the underlying molecular mechanisms have not been thoroughly ascertained. Furthermore, andrographolide seems to have pleiotropic effects on signaling pathways involved in inflammatory and oxidative stress responses, but the mechanisms underlying these effects appear different in various cell types [20, 21, 25–27]. In this study, our focus is to investigate the effects of andrographolide on HO-1 expression in astrocytes. Because HO-1 is a known gene target of transcription factor NF-E2-related factor 2 (Nrf2), which is critically involved in cellular defense against oxidative stress [11, 28, 29], we also studied andrographolide effects on astroglial Nrf2 regulation.

## Methods

### Reagents

**Fig. 1:**
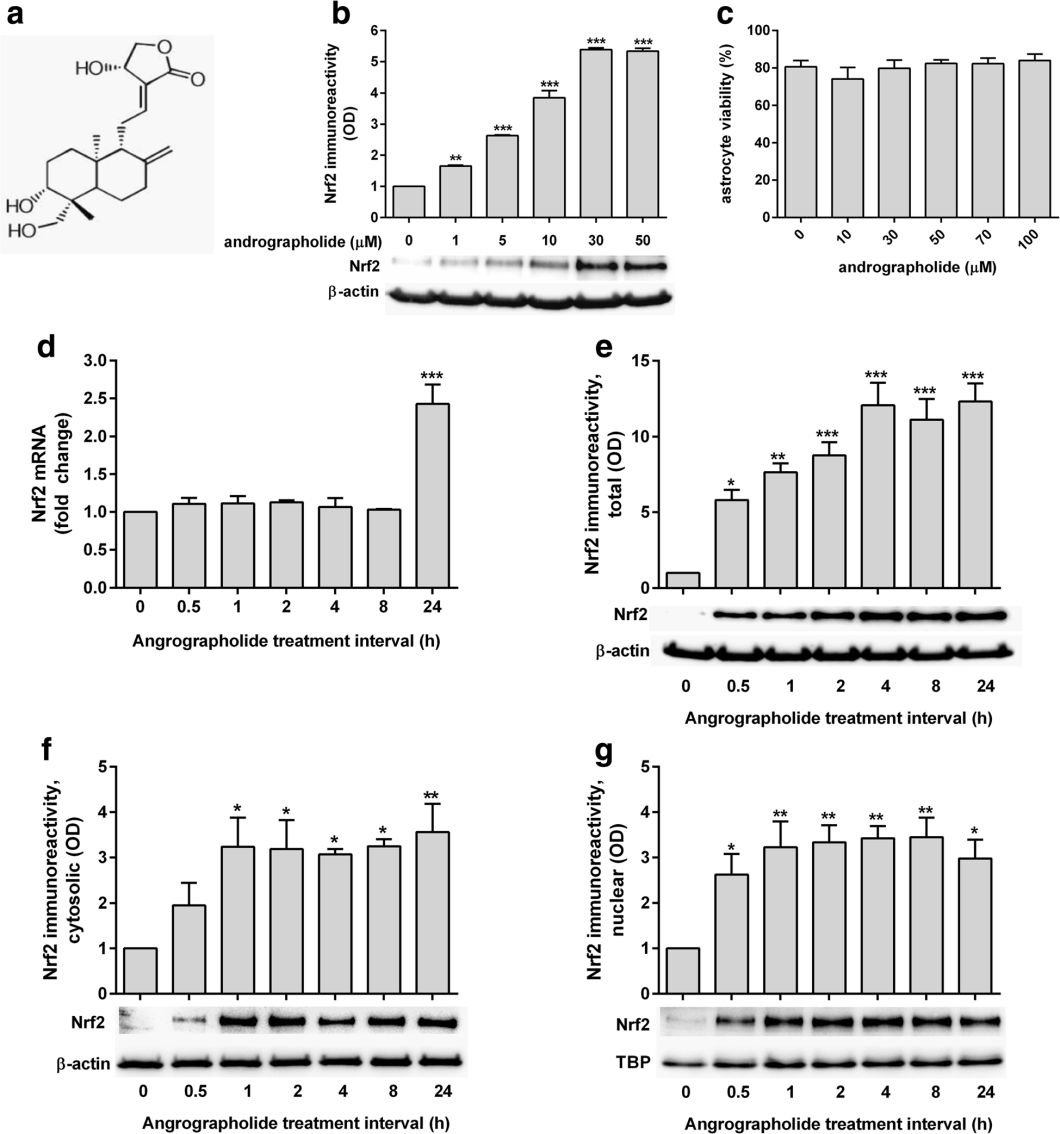
Dose and time-course of andrographolide’s effects on Nrf2. **a** Structure of andrographolide: 3-[2-[decahydro-6-hydroxy-5-(hydroxymethyl)-5,8a-dimethyl-2-methylene-1-napthalenyl]ethylidene]dihydro-4-hydroxy-2(3H)-furanone (CAS no. 5508-58-7). **b** Bar graphs depict mean ± S.E.M. Nrf2 immunoreactivities (optical density, OD fold changes normalized to β-actin) in rat primary astrocytes treated with andrographolide at the indicated concentrations for 1 h (with representative immunoblot of 3 independent experiments), with the vehicle-only (“0 μM”) group set at 1. **c** Cell viability (in mean ± S.E.M. % of vehicle-only from three independent experiments) of primary astrocytes after 24 h treatment with andrographolide at the indicated concentrations. **d** mRNA and **e** immunoreactivity changes of Nrf2 in primary astrocytes treated with andrographolide (50 μM) for the indicated time intervals, together with immunoreactivites in the **f** cytosolic and **g** nuclear fractions (with representative immunoblots). Bar graphs show mRNA or immunoreactivity expressed as mean ± S.E.M. fold change in transcript level or optical density (OD), respectively, with vehicle-only (“0 h”) group set as 1, from three to four independent experiments. Raw transcript values were normalized to mean expression of housekeeping genes (see the “Methods” section) prior to conversion to fold-change values while immunoreactivities were normalized to β-actin except for Nrf2 nuclear fractions, which were normalized to TATA-binding protein (TBP). *p < 0.05; **p < 0.01; and ***p < 0.001; significantly different from vehicle-only group (one-way ANOVA with Dunnett’s post hoc tests)

Andrographolide (≥98 % purity, see Fig. 1a), cycloheximide (CHX), and other chemical reagents were purchased from Sigma-Aldrich Ltd. (St. Louis, MO, USA) unless otherwise specified. Various concentrations of andrographolide, along with mitogen-activated protein kinase (MAPK) inhibitors SB202190 and PD98059 (Tocris Bioscience, Bristol, UK) were dissolved in up to 0.1 % dimethyl sulfoxide (DMSO) for primary astrocyte treatment. 0.1 % DMSO was also used as a control (vehicle) in the cell-based assays.

### Primary astrocyte culture

Primary astrocyte cultures for in vitro assays were obtained from newborn Sprague Dawley rat pups (postnatal days 1–3) as previously described [20]. Briefly, isolated cortices were separated from the meninges in PBS, dissociated with 0.25 % trypsin-EDTA (ThermoFisher Scientific, Waltham, MA, USA) and filtered through a 40-μm nylon cell strainer (BD Falcon, Franklin Lakes, NJ, USA). The resultant filtrate was centrifuged and resuspended in DMEM/F12 media supplemented with 10 % heat-inactivated fetal bovine serum (FBS), 100 U/mL penicillin, and 100 mg/mL streptomycin (all from ThermoFisher Scientific, Waltham, MA, USA). After 7–9 days of culture in a humidified atmosphere with 5 % CO_2_ at 37 °C, astrocytes were enriched by vigorously shaking the cell culture flasks at 350 rpm to remove non-adherent microglial cells and oligodendrocytes. Immunofluorescence staining with antibodies to glial fibrillary acidic protein indicated the purity of the resultant astrocyte cultures to be >95 % (data not shown).

### Cell viability assays

Rat primary astrocytes were plated onto 24-well tissue culture plates at a density of 1 × 10^5^ cells per well and treated with various concentrations of andrographolide (0–100 μM) for 24 h. Cell concentrations and viability (recorded as % viable cells) were determined with the Muse™ Cell Analyzer (Merck Millipore, Darmstadt, Germany) according to manufacturer’s instructions.

### Subcellular fractionation

Subcellular fractionation was performed using Nuclear Extract kit according to manufacturer’s instructions (Active Motif, Tokyo, Japan). Briefly, media was aspirated out from 10-cm culture dishes and the cells washed with 5 ml ice-cold phosphate-buffered saline (PBS) with PhosSTOP™ phosphatase inhibitor cocktail from Roche Life Science (Penzberg, Germany) and then transferred to a prechilled conical tube. Subsequently, the cell suspension was centrifuged at 200×*g* for 5 min at 4 °C, and the resulting pellet re-suspended in hypotonic buffer on ice, followed by addition of detergent and vortexing to obtain cell lysis (checked under microscope). After further centrifugation (14,000*g*, 30 s, 4 °C), the supernatant (cytoplasmic fraction) was transferred into new tubes while the pellet was re-suspended in proprietary lysis buffer. Samples were incubated for 30 min before centrifugation (14,000*g*, 10 min, 4 °C). The resulting supernatant (nuclear fraction) was transferred to new tubes and stored frozen until use.

### Real-time PCR

For measurements of Nrf2 and HO-1 messenger RNA (mRNA) expression, treated astrocytes were lysed in TRIzol® reagent (ThermoFisher Scientific, Waltham, MA, USA), precipitated, and then processed for RNA isolation according to manufacturer’s instructions (NucleoSpin® RNA kit from Macherey-Nagel GmbH, Düren, Germany). After the assessment of RNA concentration and purity with a NanoDrop spectrophotometer (ThermoFisher Scientific, Waltham, MA, USA), complementary DNA (cDNA) was synthesized from RNA samples using high-capacity cDNA reverse transcriptase kits and quantitative real-time PCR was performed using a Applied Biosystems® StepOnePlus™ Real-Time PCR instrument (ThermoFisher Scientific, Waltham, MA, USA). Table 1 lists the primer sequences of the genes of interest, and fold-change values of gene expression relative to vehicle-treated controls were computed for each experimental group using the 2^−ΔΔCT^ formula, after the normalization against the geometric mean of GAPDH and β-actin expression.

**Table 1:** Primary antibodies and primers used in this study

Target	Antibody	Format	Dilution^a^	Source Company
OH-1	OH-1	Rabbit polyclonal	1:1000; 1:200 (IF)	Abcam
Nrf2	C-20	Rabbit polyclonal	1:1000	Santa Cruz
pS40-Nrf2	phos-Nrf2	Rabbit monoclonal	1:1000	Abcam
Nqo1	C-19	Goat polyclonal	1:1000	Santa Cruz
Keap1	E-20	Goat polyclonal	1:1000	Santa Cruz
Total Erk1/2	Erk1/2	Rabbit polyclonal	1:1000	Cell Signaling
pT202/pY204-Erk	phos-Erk	Rabbit polyclonal	1:1000	Cell Signaling
Total p38	p38	Rabbit polyclonal	1:1000	Cell Signaling
pT180/pY182-p38	D3F9	Rabbit monoclonal	1:1000	Cell Signaling
β-actin	AC-15	Mouse monoclonal	1:5000	Sigma-Aldrich
Lamin B1	Lamin B1	Rabbit polyclonal	1:10,000	Abcam
TBP	TBP	Mouse monoclonal	1:5000	Abcam
Ubiquitin	P4D1	Mouse monoclonal	30 μL per 1 mg lysate protein (IP)^b^	Santa Cruz
Target	Forward primer		Reverse primer	
OH-1	5′-GGCTCTCTTTTCTTGGGCCT-3′		5′-GCCTCTACCGACCACAGTTC-3′	
Nrf2	5′-CAGTCTTCACCACCCCTGAT-3′		5′-CAGTGAGGGGATCGATGAGT-3′	
Nqo1	5′-GCGAGCGGGGAAAATACTCT-3′		5′-CCTCCTGCCCTAAACCACAG-3′	
β-actin	5′-ACCCGCGAGTACAACCTTCT-3′		5′-TTCTGACCCATACCCACCAT-3′	
GAPDH	5′-CTCATGACCACAGTCCATGC-3′		5′-TTCTGACCCATACCCACCAT-3′	

^a^Stated dilutions are for immunoblotting except for immunofluorescence (IF) staining and immunoprecipitation (IP). Source companies are Abcam (Cambridge, UK); Cell Signaling Technology (Danvers, MA, USA); Santa Cruz Biotechnology (Dallas, TX, USA); and Sigma-Aldrich (St. Louis, MO, USA)

^b^Conjugated with agarose beads (500 μg antibody/0.25 mL agarose)

### Immunofluorescence imaging

Astrocytes plated on glass coverslips were treated with andrographolide, then fixed with 4 % paraformaldehyde/PBS for 15 min, and washed thrice with PBS followed by incubation with permeabilizing buffer containing 0.1 % Triton-X100 in PBS for 5 min at 25 °C. The cells were then incubated in blocking solution (5 % BSA in permeabilizing buffer) for 1 h before incubation overnight with primary antibodies against HO-1 (1:200 dilution) in blocking solution at 4 °C. Subsequently, cells were washed thrice with PBS and incubated with anti-mouse IgG Alexa Flour® 488 (1:400 dilution, Cell Signaling Technology, Danvers, MA, USA) for 1 h at 25 °C. Cells were then washed with PBS before mounting the coverslips onto glass slides using mounting medium containing DAPI nuclear stain (Vector Laboratories, Burlingame, CA, USA). Immunofluorescence images were taken with an Axioplot microscope equipped with Carl Zeiss 510 confocal imaging scan-head and software (Carl Zeiss MicroImaging, Jena, Germany), with the same parameters used for all images.

### Immunoblotting

Astrocytes treated with andrographolide with and without chemical inhibitors were lysed in situ on tissue culture plates by adding boiling Laemmli sample buffer (Bio-Rad, Berkeley, CA, USA), heated further at 95 °C for 5 min, then allowed to cool before electrophoretic separation on 10 % polyacrylamide gels, transferred onto nitrocellulose membranes (ThermoFisher Scientific, Waltham, MA, USA), and blocked with 5 % non-fat milk in 10 mM PBS, pH7.4 with 0.1 % Tween® 20 (PBST) at room temperature for 1 h. Membranes were then washed and probed with primary antibody diluted in PBST with 5 % bovine serum albumin overnight at 4 °C. The primary antibodies used are listed in Table 1. Following primary antibody incubation, membranes were washed with PBST, then incubated with horse radish peroxidase (HRP)-conjugated secondary antibodies (goat anti-rabbit, goat anti-mouse, and donkey anti-goat, respectively, from Jackson ImmunoResearch, West Grove, PA, USA), and diluted at 1:5000 for 1 h at 25 °C. To detect the proportion of phosphorylated protein, membranes were first probed for phospho-proteins then stripped and reblotted for total proteins. Immunoblots were visualized using HRP substrate (Luminata™ Forte or Crescendo, Merck Millipore, Darmstadt, Germany) and quantified by image analyzer (UVItec Ltd., Cambridge, UK).

### Immunoprecipitation

Treated astrocytes were harvested with RIPA buffer (Santa Cruz Biotechnology, Dallas, TX, USA) supplemented with Complete ULTRA™ protease inhibitor tablets and PhosSTOP™ phosphatase inhibitor (Roche Life Science, Penzberg, Germany). The resultant lysate was sonicated and centrifuged at 14,000×*g* for 10 min at 4 °C, and supernatant was measured for protein (Pierce™ Commassie kit, ThermoFisher Scientific, Waltham, MA, USA). Input samples were prepared by adding 70 μg protein in 1:1 ratio to boiling Laemmli sample buffer with further heating at 95 °C for 5 min. For immunoprecipitation, 1 mg lysate was incubated with anti-ubiquitin monoclonal antibody-conjugated agarose beads (Santa Cruz Biotechnology, Dallas, TX, USA) for 3 h at 4 °C with rotation. Agarose beads were then pelleted and washed four times with ice-cold immunoprecipitation buffer (20 mM Tris-HCl pH 8, 140 mM NaCl, 2 mM EDTA, 1 % Triton X-100), before adding Laemmli buffer and boiling. Input samples and immunoprecipitates were electrophoretically resolved on 10 and 8 % polyacrylamide gels, respectively, then transferred onto membranes, and immunoblotted for Nrf2 (see above, and Table 1).

### Statistical analyses

Data analyses were performed using SPSS Statistics software (version 21, IBM Inc., Armonk, NY, USA). Dose effects of andrographolide were compared to untreated controls using analysis of variance (ANOVA) with Dunnett’s post hoc tests, while other pair-wise comparisons of the experimental groups were performed using ANOVA followed by Bonferroni’s post hoc tests, with *p* values <0.05 considered statistically significant. All experiments were performed independently at least three times.

## Results

### Andrographolide positively regulated Nrf2 in astrocytes

To assess the potential of andrographolide to induce Nrf2, a known regulator of HO-1 transcription [11, 28, 29], primary astrocytes were treated with various concentrations of andrographolide for 1 h and immunoblotted with Nrf2 antibody. Interestingly, while the predicted molecular weight of Nrf2 is around 66 kDa, there is increasing evidence that the biologically relevant bands fall around 95–110 kDa [30], and this unusual migration pattern of Nrf2 may be due to actin binding or the high acidic residue content of Nrf2 [31, 32]. Indeed, we observed two major bands above the 50 and 100 kDa molecular weight markers in our immunoblots (Additional File 1: Figure S1) and have selected the ~110 kDa bands for analyses. Figure 1b shows that andrographolide dose-dependently increased Nrf2 levels from as low as 1 μM, while astrocyte viability was not significantly affected with up to 100 μM andrographolide for 24 h (Fig. 1c).

### Independent time-course of andrographolide’s effects on Nrf2 mRNA versus protein in astrocytes

Next, we studied the time-course of both Nrf2 mRNA and protein changes in andrographolide-treated astrocytes. Interestingly, while treatment with andrographolide led to an upregulation of Nrf2 mRNA, the effect was not evident at 8 h and was only significant by 24 h (Fig. 1d). In contrast, Nrf2 protein levels increased significantly by 30 min and the increase was sustained throughout the study time-course (Fig. 1e), suggesting that andrographolide-mediated increases in Nrf2 protein levels at the acute stage (<8 h) did not occur via the upregulation of gene expression and de novo protein synthesis but rather may be facilitated by changes in protein level regulation, e.g., activation or turnover. Furthermore, subcellular fractionation revealed that andrographolide promoted Nrf2 accumulation in both nuclear and cytoplasmic compartments (Fig. 1f, g). Significant increases of Nrf2 in nuclear fractions were observed by 30 min which coincided with early upregulation of HO-1 mRNA level (see Fig. 3a). Taken together, these data indicate that andrographolide could induce HO-1 expression acutely by promoting the accumulation of Nrf2.

### Andrographolide has no effect on Nrf2 phosphorylation or Keap1

**Fig. 2:**
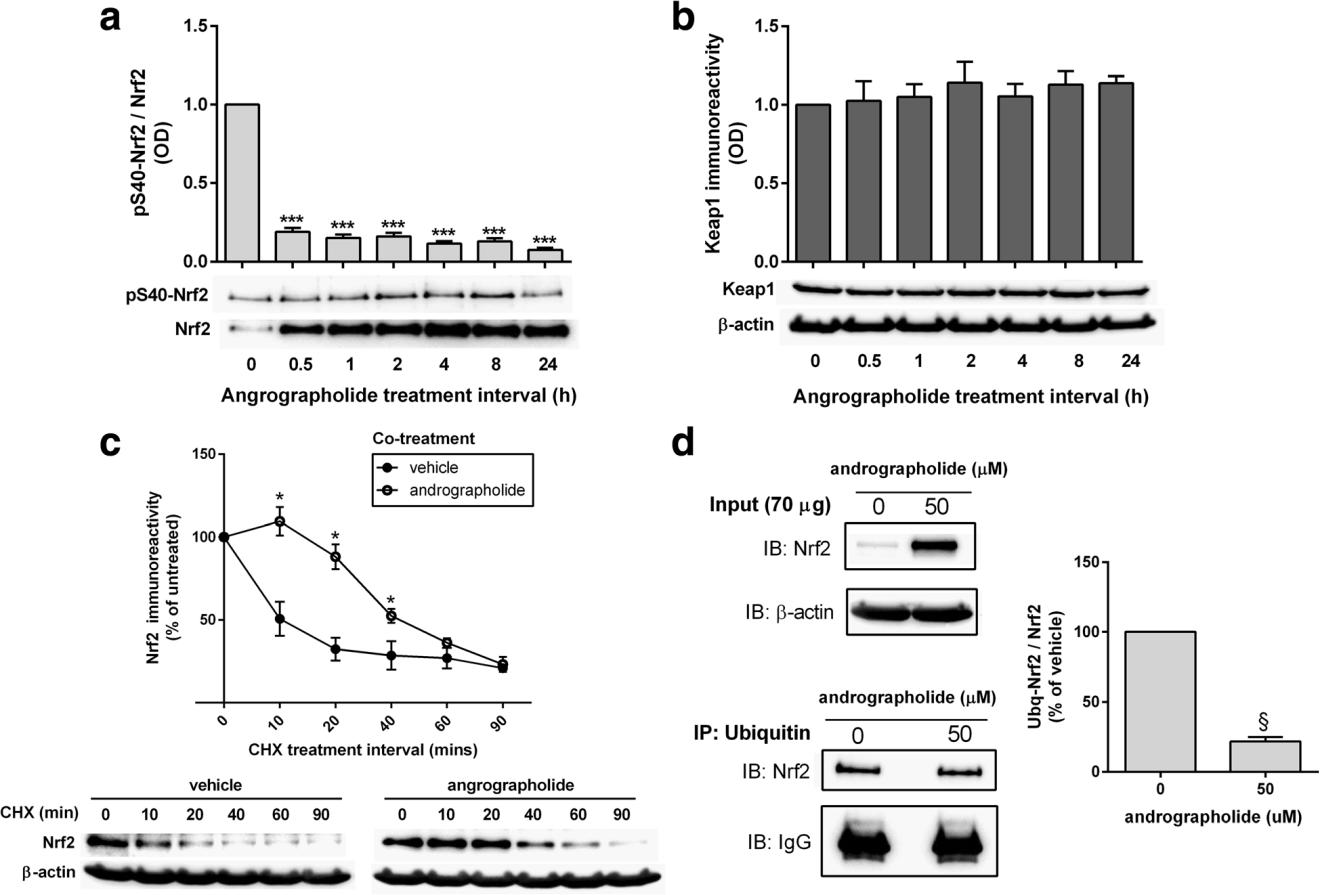
Andrographolide increases stability of Nrf2 protein by altering ubiquitination efficiency. Primary astrocytes were treated with andrographolide (50 μM) for the indicated time intervals and measured for immunoreactivities of **a** pSer40 Nrf2/total Nrf2 as well as **b** Keap1 immunoreactivity normalized to β-actin (with representative immunoblots), and bar graphs showing mean ± S.E.M. fold changes in optical density (OD) with vehicle-only (“0 h”) group set as 1. ***p < 0.001; significantly different from vehicle-only group (one-way ANOVA with Dunnett’s post hoc tests). **c** Rat primary astrocytes were treated with cycloheximide (CHX, 10 μg/mL) with or without andrographolide (50 μM) co-incubation for the indicated time intervals and then measured for total Nrf2 immunoreactivity (with representative immunoblots). The graph represents mean ± S.E.M. Nrf2 immunoreactivities in vehicle-only (*filled circles*) and andrographolide co-treated (*open circles*) groups expressed as % of untreated (“0 h” CHX) group. *p < 0.05; significantly different from vehicle-only group (Student’s *t* tests). **d** Rat primary astrocytes were incubated with or without 50 μM of andrographolide for 1 h and processed for immunoprecipitation (see the “Methods” section), with representative input (IB) and ubiquitin-immunoprecipated (IP) blots for Nrf2. Bar graph shows mean ± S.E.M. immunoreactivity (fold changes in optical densities, OD with untreated group set at 100 %) of Ub-Nrf2/total Nrf2 normalized to β-actin. ^§^ *p* < 0.001 for comparison with untreated control (Student’s *t* tests). All data were from three to four independent experiments

One of the key regulators of Nrf2 is Keap1, which binds to Nrf2 and promotes its ubiquitination and subsequent degradation by 26S proteasome [33]. Phosphorylation of Nrf2 at Ser40 by protein kinase C is known to disrupt Nrf2 binding to Keap1, leading to nuclear accumulation of Nrf2 [34, 35]. However, while no significant change in pSer40 Nrf2 immunoreactivity was found, the total Nrf2 increased with andrographolide treatment, thus resulting in a significantly decreased ratio of pSer40 Nrf2 to total Nrf2 (Fig. 2a). The possibility of andrographolide promoting Nrf2 protein accumulation by altering Keap1 levels was also considered; however, Fig. 2b showed no change in Keap1 immunoreactivity with andrographolide treatment. Taken together, the data suggest that andrographolide did not affect Nrf2 accumulation by either Ser40 phosphorylation-mediated escape from Keap1 or changes to Keap1 itself.

### Andrographolide reduced Nrf2 turnover and ubiquitination

We next studied effects of andrographolide on Nrf2 turnover rate. Treatment of astrocytes with cycloheximide (CHX), which inhibits de novo protein synthesis, showed that Nrf2 normally had a high turnover rate (half-life of around 10 min, Fig. 2c), in agreement with previous observations [36]. With andrographolide co-treatment, however, the turnover of Nrf2 was significantly decreased, with half-life increased to around 40 min (Fig. 2c). Since ubiquitination signals for protein degradation, the effect of andrographolide on levels of ubiquitinated Nrf2 was assessed. Interestingly, ubiquitin immunoprecipitation showed higher total Nrf2 (input protein) immunoreactivity without proportional increases in ubiquitinated Nrf2 (Fig. 2d), thus suggesting that altered Nrf2 ubiquitin efficiency may be one possible mechanism by which andrographolide increased Nrf2 stability and subsequent upregulation of effector genes, including HO-1.

### Andrographolide positively regulated HO-1 in astrocytes

**Fig. 3:**
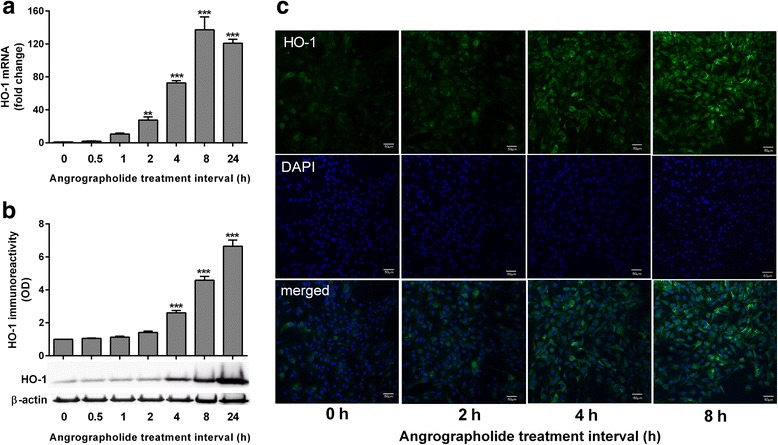
Andrographolide upregulates heme oxygenase-1 in astrocytes. Primary astrocytes were treated with andrographolide (50 μM) for the indicated time intervals and measured for HO-1 **a** mRNA and **b** immunoreactivity (with representative immunoblots), together with respective bar graphs of mean ± S.E.M. fold changes in transcript level or optical density (OD), with vehicle-only (“0 h”) group set as 1, from three to four independent experiments. Raw transcript values were normalized to mean expression of housekeeping genes (see the “Methods” section) prior to conversion to fold-change values while HO-1 immunoreactivity was normalized to β-actin. **p < 0.01 and ***p < 0.001; significantly different from vehicle-only group (one-way ANOVA with Dunnett’s post hoc tests). **c** Primary astrocytes treated with 50 μM andrographolide for the indicated time intervals were processed for HO-1 immunofluorescence staining using Alexa Flour® 488-conjugated secondary antibody (*green*), while DAPI counter stain (*blue*) was used to visualize cell nuclei. *Scale bars* = 50 μm

As Nrf2 levels increased within 1 h of andrographolide treatment, and therefore likely to regulate HO-1 acutely, we performed RT-PCR and immunoblot time-course experiments and showed that andrographolide (50 μM) elevated HO-1 mRNA and protein in a time-dependent manner, with mRNA increase reaching statistical significance from 2 h while increase in protein was statistically significant from 4 h (Fig. 3a, b). These data were corroborated qualitatively by immunofluorescence staining which showed increased HO-1 immunoreactivity from around 4 h after andrographolide treatment (Fig. 3c). Therefore, andrographolide’s effects on HO-1 expression may be mediated via a relatively rapid (<1 h) upregulation of Nrf2. As andrographolide’s effects on antioxidative pathways are unlikely to be restricted to HO-1 given Nrf’s regulation of multiple gene targets [29], we examined another detoxification and antioxidant molecule, NAD(P)H quinone oxoreductase (Nqo1) [11] and found, indeed, that andrographolide treatment also increased its expression (Additional File 2: Figure S2). This suggests that andrographolide’s antioxidant effect is unlikely to be mediated by the upregulation of HO-1 only, and other antioxidant molecules such as Nqo1 are also involved.

### Upregulation of Nrf2 and HO-1 by andrographolide is mediated by p38 MAPK- and ERK-dependent pathways

**Fig. 4:**
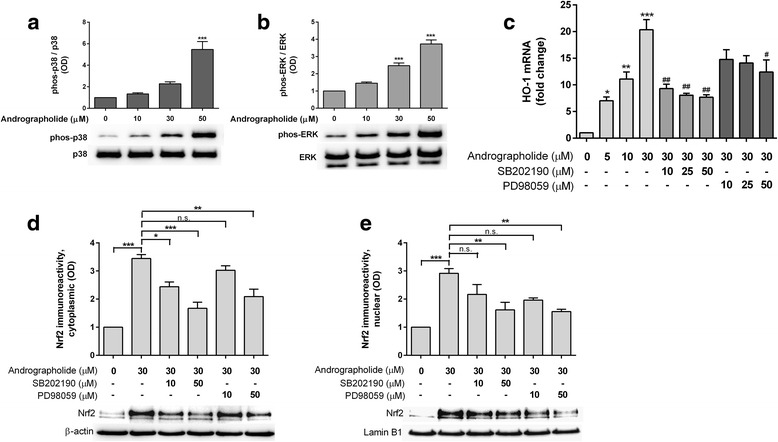
Andrographolide induces HO-1 expression through p38 MAPK- and ERK-dependent regulation of Nrf2. Effects of andrographolide on **a** p38 MAPK and **b** ERK activation. Primary astrocytes were treated with increasing concentrations of andrographolide for 6 h and processed for immunobloting. Bar graphs showing mean ± S.E.M. fold changes in optical density (OD, with vehicle-only “0 μM” group set as 1) of phospho-protein normalized to total protein, ***p < 0.001; significantly different from vehicle-only group (one-way ANOVA with Dunnett’s post hoc tests). **c** Effects of p38 MAPK and ERK inhibition on HO-1 mRNA expression. Primary astrocytes were pretreated with or without SB202190 (p38 MAPK inhibitor) or PD98059 (MEK/ERK inhibitor) for an hour followed by 4 h of incubation with andrographolide (with presence of inhibitors) before processing for RT-PCR. Bar graphs are mean ± S.E.M. fold change in transcript level, with untreated group set as 1. Raw transcript values were normalized to mean expression of housekeeping genes (see the “Methods” section) prior to conversion to fold-change values. *p < 0.05, **p < 0.01, and ***p < 0.001; significantly difference from untreated group (one-way ANOVA followed by Dunnett’s post hoc tests.). ^#^ *p* < 0.05, ^##^ *p* < 0.01; significantly different from 30 μM andrographolide (one- way ANOVA followed by Bonferroni’s post hoc tests). **d**, **e** Effects of p38 MAPK and ERK inhibition on Nrf2 in the cytosolic and nuclear compartments, respectively. Primary astrocytes were pretreated with PD98059 or SB202190 for 1 h followed by another 1 h of incubation with 30 μM andrographolide (in the presence of inhibitors). Cytoplasmic and nuclear fractions were separated and processed for immunoblotting. Bar graphs show immunoreactivities (mean ± S.E.M. fold changes in optical densities, OD, with untreated group set at 1) of Nrf2 normalized to β-actin (cytoplasmic) or to lamin B1 (nuclear). *p < 0.05, **p < 0.01, ***p < 0.001; significant pairwise difference and n.s. = not significant (*p* > 0.05) using one-way ANOVA with Bonferroni’s post hoc tests. All data were from three to four independent experiments

Mitogen-activated protein kinases (MAPKs) such as p38 and extracellular signal-regulated kinase (ERK) are known to facilitate Nrf2 activation and increased expression of Nrf2 target genes [37–40], which led us to speculate whether andrographolide signals to these MAPKs. Interestingly, andrograpolide dose-dependently increased immunoreactivities of phosphorylated (activated) p38 MAPK (Fig. 4a) and p42 ERK (Fig. 4b). Furthermore, pretreatment with inhibitors of p38 MAPK (SB202190) or ERK (PD98059) partially attenuated the upregulation of HO-1 mRNA by andrographolide (Fig. 4c). Similarly, andrographolide-induced Nrf2 accumulation in both cytoplasmic and nuclear fractions was partially attenuated by pretreatment with SB202190 and PD98059 (Figs. 4d, e). These results support the involvement of p38 MAPK and ERK signaling in regulating andrographolide’s effects on Nrf2 and HO-1.

## Discussion

Neuroinflammation and oxidative stress are highly interconnected processes, and dysregulation of these processes may be pathogenic in various neuroinflammatory and neurodegenerative conditions, leading to recent research efforts to uncover and assess the therapeutic potential of anti-neuroinflammatory and antioxidative compounds [1–5, 11, 41]. To this end, we have previously shown that the anti-neuroinflammatory effects of andrographolide on astrocytes are mediated via the inhibition of NF-kB and c-Jun N-terminal kinase [20, 21]. In this study, our data suggest that andrographolide may also have antioxidative effects in primary astrocytes via the upregulation of Nrf2, a master regulator of antioxidant responses [29]. In support of this postulate, a previous screening of 54 bioactive compounds identified andrographolide as a more potent Nrf2 activator than tert-butylhydroquinone (tBHQ), an antioxidant frequently used to study Nrf2/ARE activation [42]. Furthermore, we found that HO-1, a known target of Nrf2-regulated transcription and an antioxidative, cytoprotective protein in both CNS and non-CNS cells [8–10], was also upregulated in andrographolide-treated astrocytes. Here, we showed, remarkably, that increases of HO-1 mRNA were observable within 1 h of andrographolide incubation, closely following the rapid accumulation (within 30 min) of Nrf2 (Figs. 1 and 3). We subsequently found that Nrf2 increases were due to reduced Nrf2 ubiquitination efficiency and turnover (Fig. 2). Taken together with observations of increased Nrf2 mRNA from between 8 to 24 h (Fig. 1a), our data suggest that andrographolide’s effect on Nrf2 is biphasic, with an acute effect on protein turnover and a more intermediate effect at the transcript level. Furthermore, while we focused on HO-1 in this study due to its established anti-neuroinflammatory and neuroprotective properties in the CNS [8, 10], it is very likely that andrographolide will upregulate other antioxidant pathways and molecules via Nrf2 [29], such as Nqo1, another molecule important in the detoxification and prevention of reactive oxygen radical formation (Additional File 2: Figure S2).

Given that the high turnover rate of Nrf2 allows it to be maintained at low, basal levels but be rapidly upregulated in response to oxidative insults [33, 36, 38], we then studied potential mechanisms underlying andrographolide’s effect on Nrf2 turnover, one of which is interaction with Keap1. Under basal conditions, Keap1 binds to the Neh2 domain of Nrf2 and sequesters it in the cytoplasm, acting as a substrate adapter for cullin-3 (Cul3) which, together with other proteins, forms an E3 ubiquitin ligase complex to promote Nrf2 ubiquitination and degradation [33]. Therefore, Nrf2 may potentially escape Keap1-mediated degradation either by downregulating Keap1 or by phosphorylation of Nrf2 at Ser40 which disrupts binding to Keap1 [34, 35]. Although our finding of unchanged Keap1 and reduced rather than increased proportion of pSer40 Nrf2 (Fig. 2a, b) suggested a Keap1-independent mechanism, we cannot rule out the possibility that andrographolide may alter Keap1-Nrf2 association by forming adducts with the thiol groups of reactive cysteine residues on Keap1, similar to that reported for other Nrf2 inducers [43]. Therefore, confirmatory studies of andrographolide’s effects on Keap1 are required.

Nrf2 is an acidic protein with ~16 % of its total amino acids made up of serine, threonine, and tyrosine residues, making it a probable substrate for several signaling kinases [44]. Emerging evidence indicate that Nrf2 phosphorylation positively regulates its stability, as treatment with phosphatase inhibitors led to Nrf2 hyperphosphorylation, accumulation, and activation of ARE-mediated reporter gene [38]. Moreover, tBHQ and other inducers increased Nrf2 stability and transactivation activity through p38 MAPK and ERK [37–39]. Similarly, we found that andrographolide activated p38 MAPK and ERK in a dose-dependent manner (Fig. 4a, b), while inhibition of p38 MAPK and ERK attenuated andrographolide-mediated upregulation of HO-1 transcription as well as Nrf2 accumulation in both cytoplasmic and nuclear compartments (Figs. 4c–e). Our data therefore suggest regulatory roles of ERK and p38 MAPK signaling in Nrf2 activation and HO-1 expression in astrocytes. It is worth noting, however, that Nrf2 and HO-1 induction were only partially attenuated by ERK and p38 MAPK inhibition (Figs. 4c–e), suggesting the involvement of other signaling molecules and pathways. Interestingly, while the two main members of ERK, p42 ERK2, and p44 ERK1 are usually co-regulated [45], andrographolide seemed to only activate p42 ERK2 (Fig. 4b), and the mechanism underlying this specificity is unclear at present. Furthermore, there are conflicting reports of andrographolide’s effects on signaling kinases, which seemed to depend at least in part on the cell types and time-course studied. For example, we have found that andrographolide inhibits JNK but activates ERK and p38 MAPK in primary astrocytes (see [21] and this study). In contrast, Lee et al. [26] showed in human hepatoma cells that p38, but not ERK, mediated the upregulation of Nrf2 and HO-1 by andrographolide, while in endothelial cells, although andrographolide activated ERK, the induction of HO-1 was dependent on phosphatidylinositol-3 kinase/protein kinase B, rather than on ERK [27]. In the case of the hepatoma cell study [26], the absence of ERK activation may be explained by a relatively short time-course (2 h), while our time-course experiments found that ERK phosphorylation was only evident after 4 h of incubation (data not shown). These data suggest that the molecular mechanisms underlying andrographolide’s effects are not generalizable across cell types, and studies are needed to characterize different cells/tissues/systems of interest. Lastly, it is at present unclear whether the various signaling kinases activate Nrf2 by direct phosphorylation, or via other signaling molecules or mechanisms. For example, p38 MAPK and ERK can inhibit glycogen synthase kinase-3β (GSK-3β) phosphorylation of the Neh6 domain of Nrf2, and as a result, attenuate Keap1-independent Nrf2 ubiquitination and degradation via the β-TrCP/Cul1 E3 ubiquitin ligase complex [44, 46–48]. Further studies are needed to investigate whether this pathway is relevant for astrocytes and other CNS cell types, including microglia and neurons treated with andrographolide. These follow-up studies are especially relevant for microglia, whose activation time-scale is faster than astrocytes and provide the initial neuroinflammatory signals which subsequently activate astrocytes [49], and therefore also represent an essential target in anti-neuroinflammatory therapeutics.

**Fig. 5:**
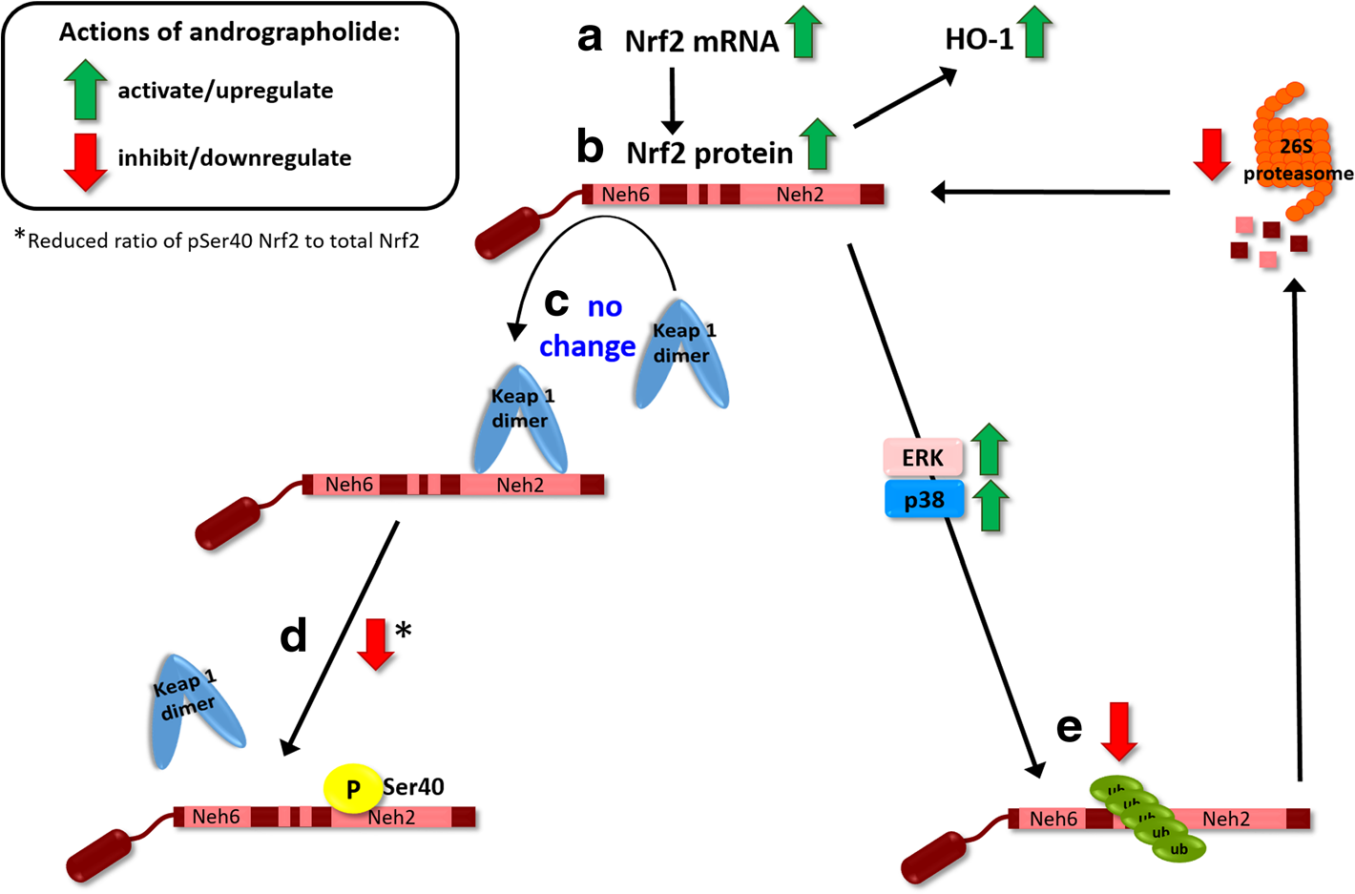
Andrographolide’s effects on Nrf2 and HO-1 in astrocytes. Summary schematic of findings of the current study. *Black solid arrows* indicate positive regulation, while activation effects of andrographolide are denoted by *green arrows*, and inhibitory effects are denoted by *red arrows. a* Andrographolide activates Nrf2 transcription between 8 and 24 h, leading to *b* increased Nrf2 proteins, which may also accumulate rapidly (within 1 h) through a process of altered ubiquitination which *c* does not alter Keap1 levels and *d* does not disrupt Nrf2-Keap1 binding through Ser40 phosphorylation within the Nrf2 Neh2 domain. Instead, andrographolide treatment leads to *e* reduced Nrf2 ubiquitination efficiency and subsequent 26S proteasomal turnover via p38 MAPK- and ERK-dependent pathways, although it is unclear whether the kinases directly act on Nrf2, or through regulation of other signaling molecules, for, e.g., GSK-3β. Further studies are required

## Conclusions

We showed in this study that andrographolide induces the master antioxidant regulator, Nrf2, as well as one of its target genes, HO-1, in primary astrocytes. The induction of Nrf2 seems to be biphasic, with an acute effect (within 1 h) of reducing Nrf2 ubiquitination efficiency and subsequent turnover and a longer term effect (between 8 and 24 h) of upregulating Nrf2 mRNA, thus enabling the rapid and sustained induction of HO-1. Furthermore, the acute effects did not seem to affect Keap1 levels but may rather be partly dependent on p38 MAPK- and ERK-mediated signaling (Fig. 5). Given the important function of astrocytes in mediating neuroinflammatory responses as well as maintaining redox homeostasis in the neuronal environment, and taking into consideration our previous work on the anti-neuroinflammatory effects of andrgrapholide, the current data provide further insights into the mechanisms underlying the pleiotropic effects of andrographolide on astrocyte-mediated antioxidant and anti-inflammatory responses, and further support the potential therapeutic utility of andrographolide for neurological conditions characterized by inflammation and oxidative stress. However, follow-up studies are needed to (i) further characterize the molecular mechanisms and signaling pathways underlying andrographolide’s induction of Nrf2, both acutely and in the longer term; (ii) uncover other antioxidant pathways and molecules which may be affected by andrograpolide; and (iii) study the effects of andrographolide in different CNS cell types, including microglia and neurons, as well as in animal models of diseases.

## Additional files

Additional file 1: Figure S1. Representative immunoblots of Nrf2 in primary astrocytes. **a** An example of time-point experiment after andrographolide treatment, nuclei fraction (Fig. 1g) and **b** an example of ubiquitin immunoprecipitation (IP) after andrographolide treatment (Fig. 2d) with input lysate on the right and IP blot on the left, with indicated molecular weight marker positions. In most cases, two prominent bands above 50 and 100 kDa were visible, and blue arrows indicate the bands selected for analyses (around 110 kDa) in accordance with Lau et al. [30]. (DOCX 431 kb) Additional file 2: Figure S2. Andrographolide upregulates NAD(P)H quinone oxoreductase 1 (Nqo1) in astrocytes. Primary astrocytes were treated with andrographolide (50 μM) for the indicated time intervals and measured for Nqo1 **a** mRNA and **b** immunoreactivity (with representative immunoblots), together with respective bar graphs of mean ± S.E.M. fold changes in transcript level or optical density (OD), with vehicle-only (“0 h”) group set as 1, from 4 independent experiments. Raw transcript values were normalized to mean expression of housekeeping genes (see the “Methods” section) prior to conversion to fold-change values while Nqo1 immunoreactivity was normalized to β-actin. ****p* < 0.001; significantly different from vehicle-only group (one-way ANOVA with Dunnett’s post hoc tests). (DOCX 176 kb)

## Abbreviations

ANOVA: Analysis of variance
ARE: Antioxidant response element
CHX: Cycloheximide
DAPI: 4′,6-Diamidino-2-phenylindole
DMEM: Dulbecco’s modified Eagle medium
DMSO: Dimethyl sulfoxide
EDTA: Ethylenediaminetetraacetic acid
ERK: Extracellular signal-regulated kinase
FBS: Fetal bovine serum
GAPDH: Glyceraldehyde-3-phosphate dehydrogenase
HO-1: Heme oxygenase-1
HRP: Horse radish peroxidase
IB: Immunoblot
IP: Immunoprecipitation
Keap1: Kelch-like ECH-associated protein 1
NF-kB: Nuclear factor-kB
Nqo1: NAD(P)H quinone oxoreductase 1
Nrf2: Nuclear factor erythroid 2-related factor 2
OD: Optical density
PBS: Phosphate-buffered saline
RT-PCR: Reverse transcription polymerase chain reaction
TBP: TATA-binding protein
