# Supplementary material for: Andrographolide induces Nrf2 and heme oxygenase 1 in astrocytes by activating p38 MAPK and ERK
Source: J Neuroinflammation. 2016 Sep 23;13:251. doi: 10.1186/s12974-016-0723-3 (PMC5034653; doi:10.1186/s12974-016-0723-3)
Supplement: Additional file 1: Figure S1. — Representative immunoblots of Nrf2 in primary astrocytes. a An example of time-point experiment after andrographolide treatment, nuclei fraction (Fig. 1g) and b an example of ubiquitin immunoprecipitation (IP) after andrographolide treatment (Fig. 2d) with input lysate on the right and IP blot on the left, with indicated molecular weight marker positions. In most cases, two prominent bands above 50 and 100 kDa were visible, and blue arrows indicate the bands selected for analyses (around 110 kDa) in accordance with Lau et al. [30]. (DOCX 431 kb) [file 12974_2016_723_MOESM1_ESM.docx]

*Additional File 1: Supplementary Figure 1*

Representative immunoblots of Nrf2 in primary astrocytes

**
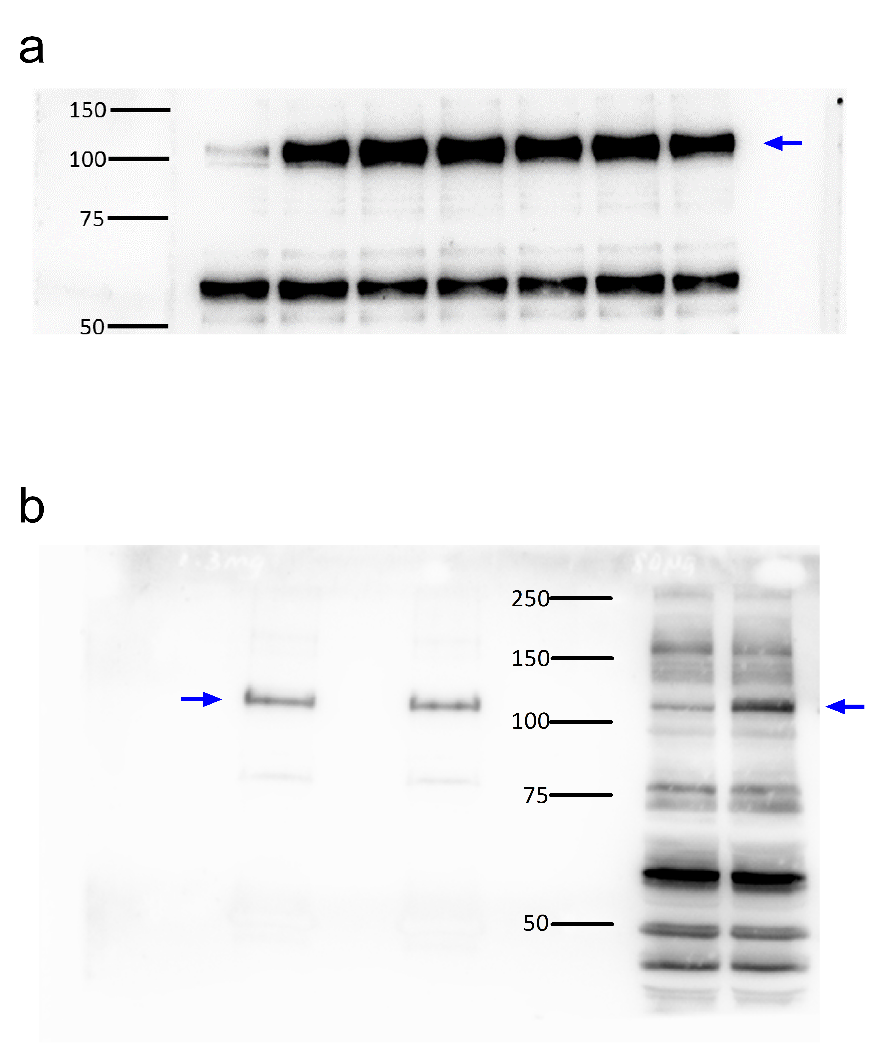
**

**Fig. S1** Representative immunoblots of Nrf2 in primary astrocytes. **a** An example of time-point experiment after andrographolide treatment, nuclei fraction (Fig. 1g) and **b** an example of ubiquitin immunoprecipitation (IP) after andrographolide treatment (Fig. 2d) with input lysate on the right and IP blot on the left, with indicated molecular weight marker positions. In most cases, two prominent bands above 50 kDa and 100 kDa were visible, and blue arrows indicate the bands selected for analyses (around 110 kDa) in accordance with Lau et al. [30].

**Wong *et al.* Andrographolide induces Nrf2 and heme-oxygenase 1 in astrocytes by activating p38 MAPK and ERK**
