# Supplementary material for: Andrographolide induces Nrf2 and heme oxygenase 1 in astrocytes by activating p38 MAPK and ERK
Source: J Neuroinflammation. 2016 Sep 23;13:251. doi: 10.1186/s12974-016-0723-3 (PMC5034653; doi:10.1186/s12974-016-0723-3)
Supplement: Additional file 2: Figure S2. — Andrographolide upregulates NAD(P)H quinone oxoreductase 1 (Nqo1) in astrocytes. Primary astrocytes were treated with andrographolide (50 μM) for the indicated time intervals and measured for Nqo1 a mRNA and b immunoreactivity (with representative immunoblots), together with respective bar graphs of mean ± S.E.M. fold changes in transcript level or optical density (OD), with vehicle-only (“0 h”) group set as 1, from 4 independent experiments. Raw transcript values were normalized to mean expression of housekeeping genes (see the “Methods” section) prior to conversion to fold-change values while Nqo1 immunoreactivity was normalized to β-actin. ***p < 0.001; significantly different from vehicle-only group (one-way ANOVA with Dunnett’s post hoc tests). (DOCX 176 kb) [file 12974_2016_723_MOESM2_ESM.docx]

*Additional File 2: Supplementary Figure 2*

Andrographolide up-regulates NAD(P)H quinone oxoreductase 1 (Nqo1) in astrocytes

**
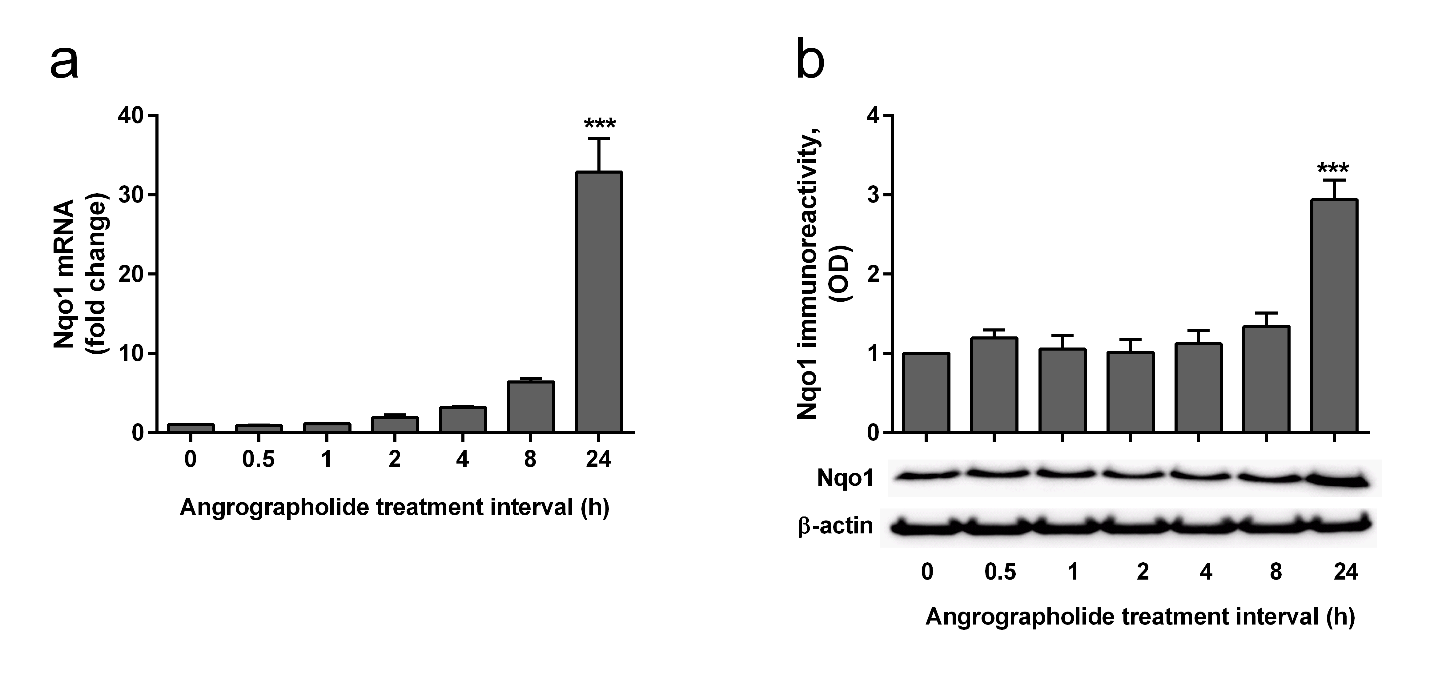
**

**Fig. S2** Primary astrocytes were treated with andrographolide (50 μM) for the indicated time intervals and measured for Nqo1 **a** mRNA and **b** immunoreactivity (with representative immunoblots), together with respective bar graphs of mean ± S.E.M. fold changes in transcript level or optical density (OD), with vehicle-only (“0 h”) group set as 1, from 3 independent experiments. Raw transcript values were normalized to mean expression of housekeeping genes (see Methods) prior to conversion to fold-change values while Nqo1 immunoreactivity was normalized to β-actin. ****p* < 0.001; significantly different from vehicle-only group (one-way ANOVA with Dunnett’s *post-hoc* tests).
